# Supplementary figures and images for: Age-dependent H3K9 trimethylation by dSetdb1 impairs mitochondrial UPR leading to degeneration of olfactory neurons and loss of olfactory function in Drosophila
Source: eLife. 2026 Mar 26;15:e103118. doi: 10.7554/eLife.103118 (PMC13155754; doi:10.7554/eLife.103118)

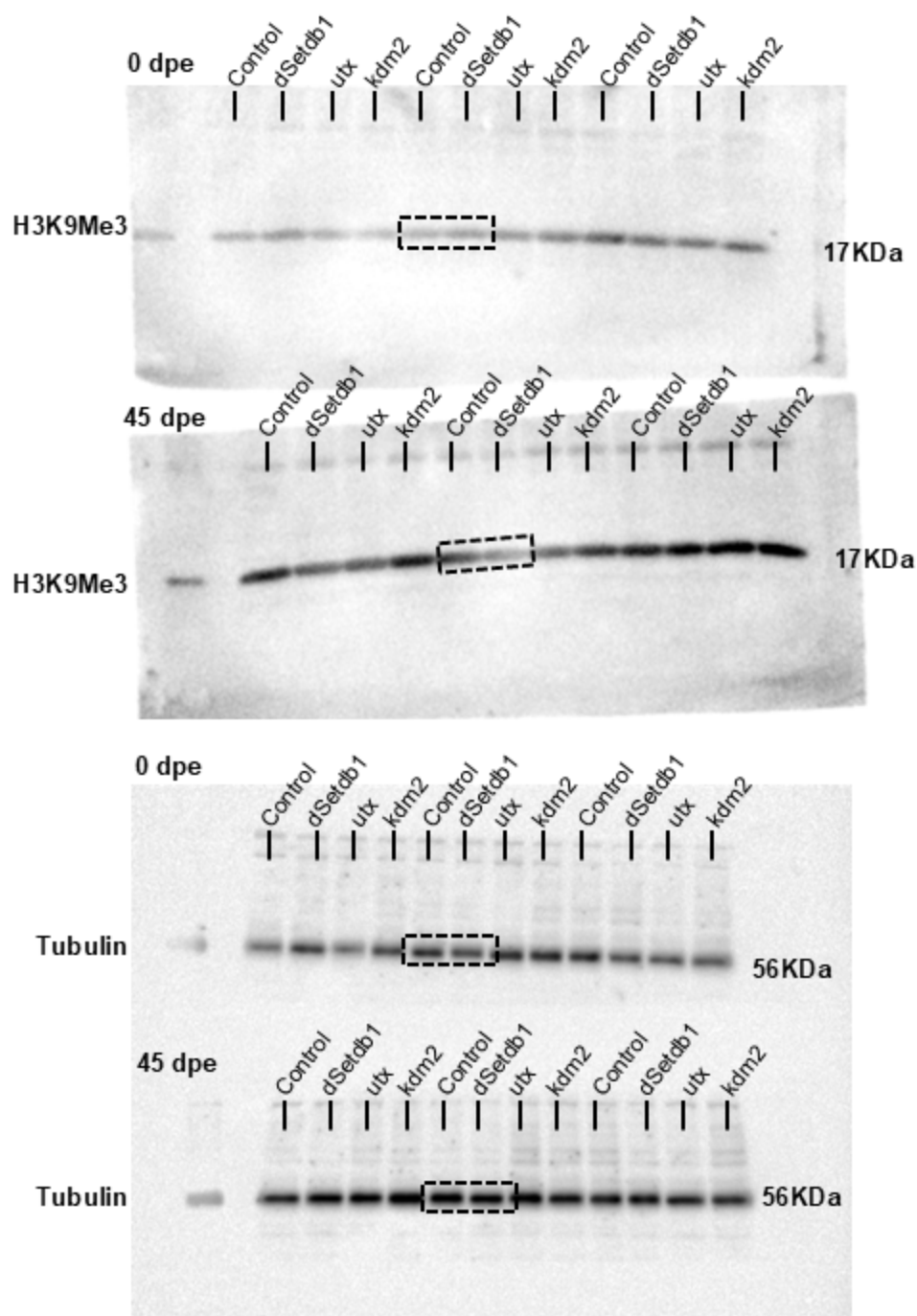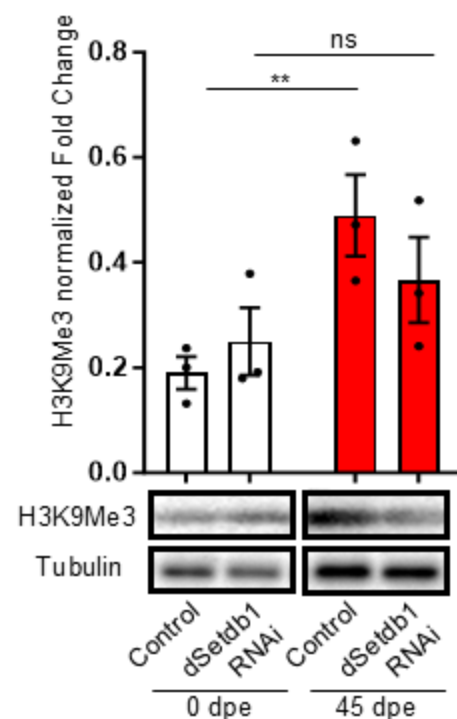

Figure 2 - source data 1  
Labelled blots

Supplement: Figure 2—source data 1. [file elife-103118-fig2-data1.zip › Figure 2-source data 1/Figure 2A Source Data 1 - Labelled blots.pdf]

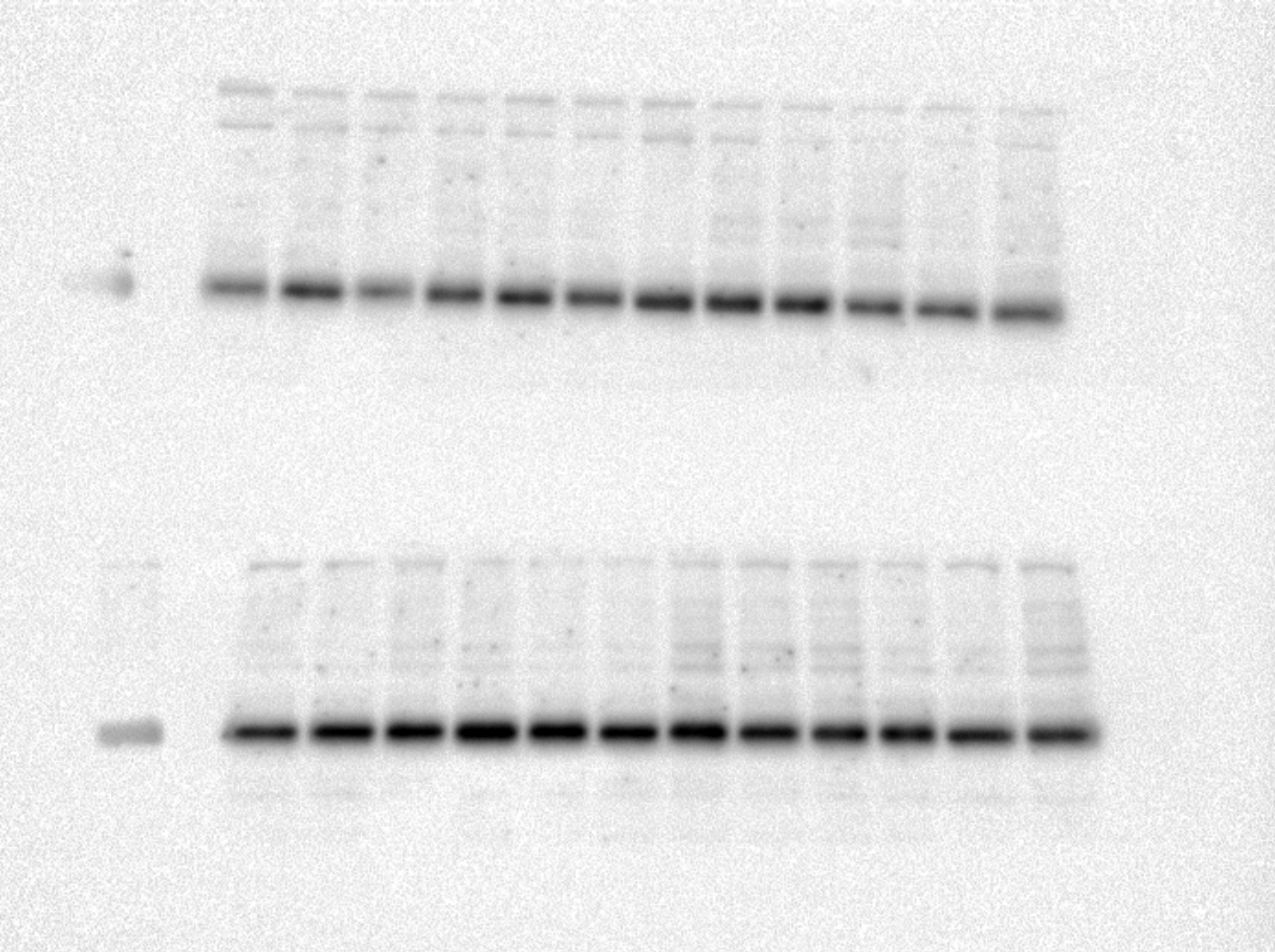

Supplement: Figure 2—source data 2. [file elife-103118-fig2-data2.zip › Figure 2-source data 2/Figure 2A Source Data 2 - Tubulin.tif]

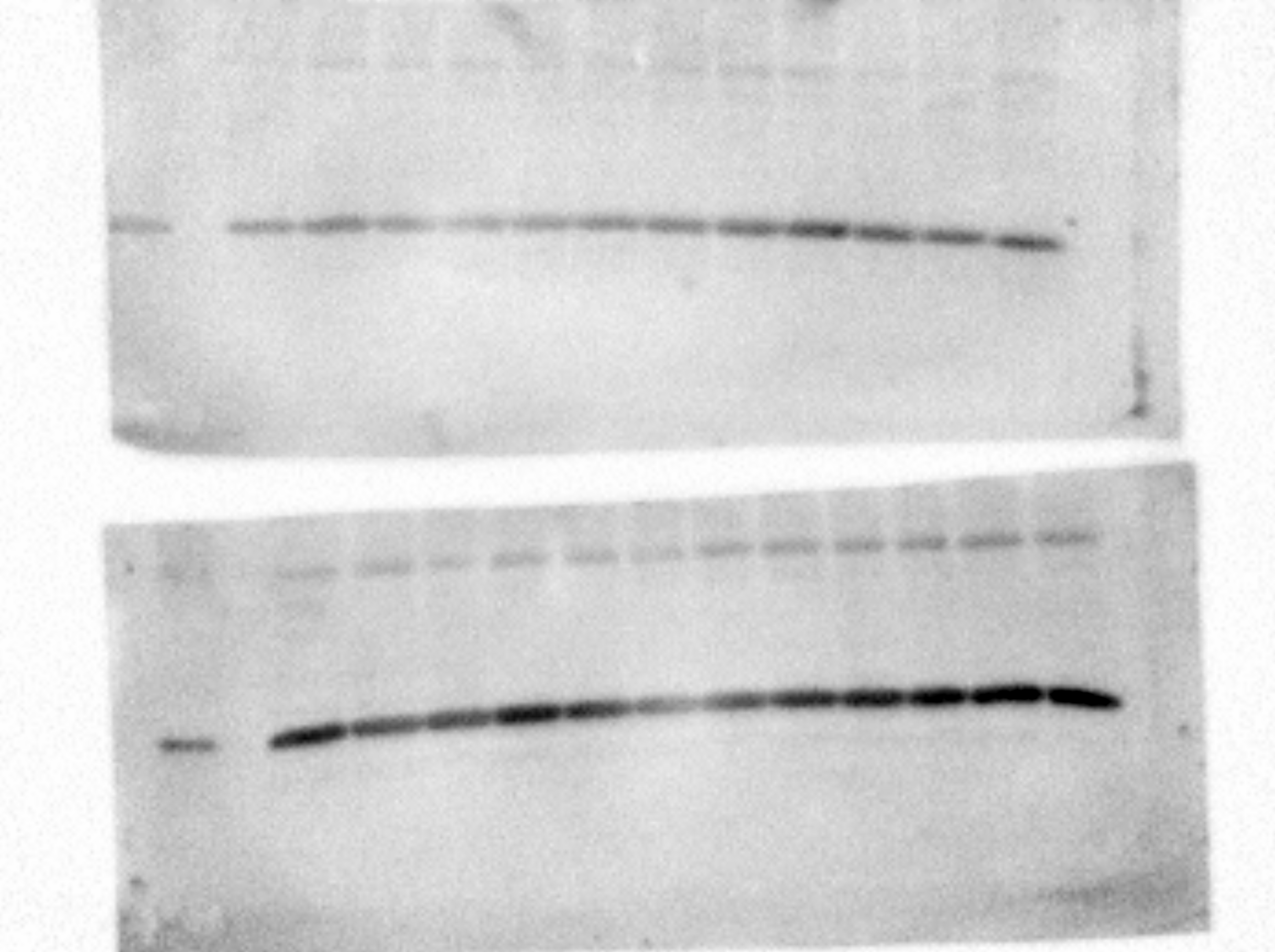

Supplement: Figure 2—source data 2. [file elife-103118-fig2-data2.zip › Figure 2-source data 2/Figure 2A Source Data 2 - H3K9Me3.tif]

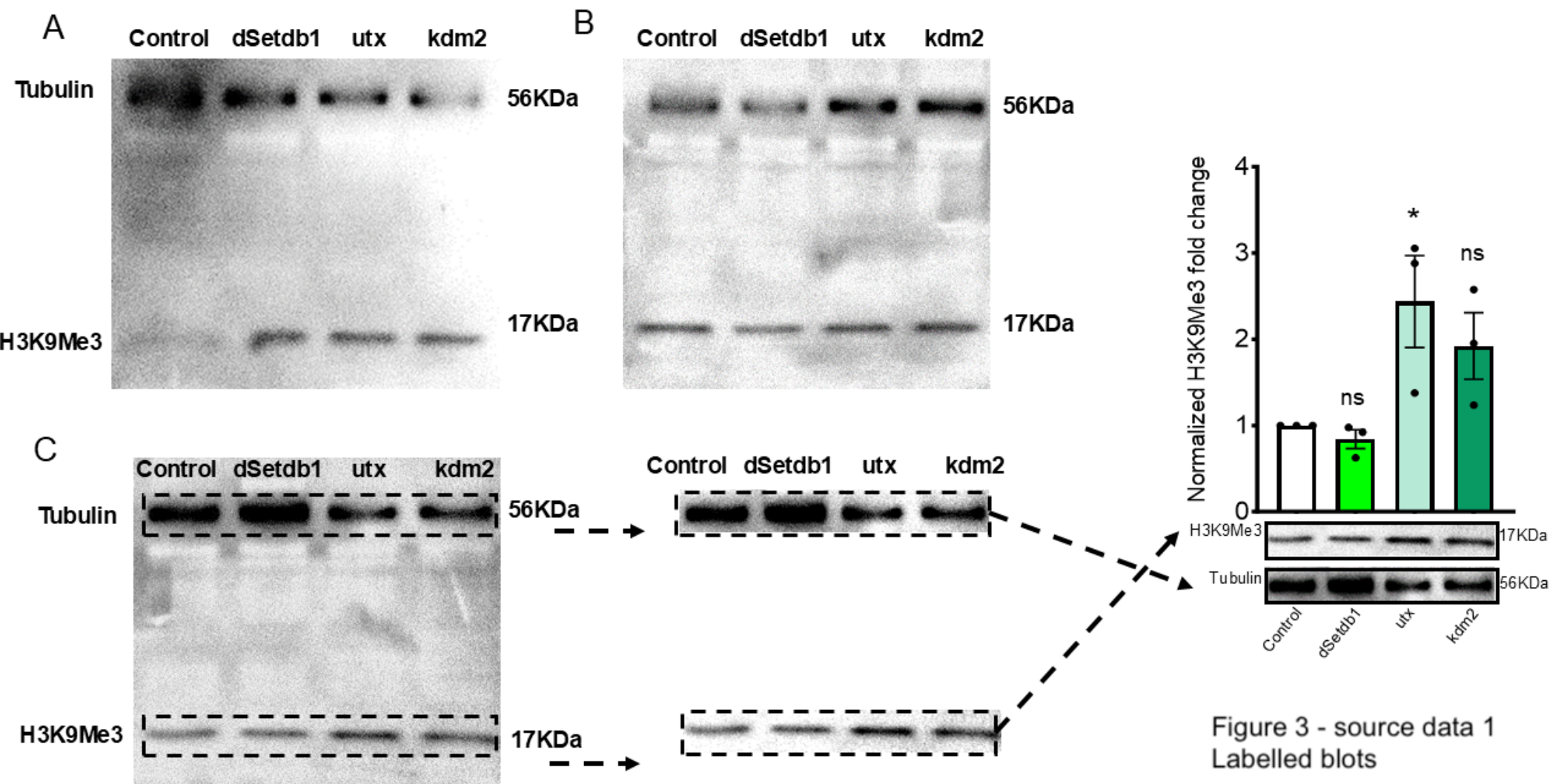

Supplement: Figure 3—source data 1. [file elife-103118-fig3-data1.zip › Figure 3-source data 1/Figure 3D Source Data 1 - Labelled blots.pdf]

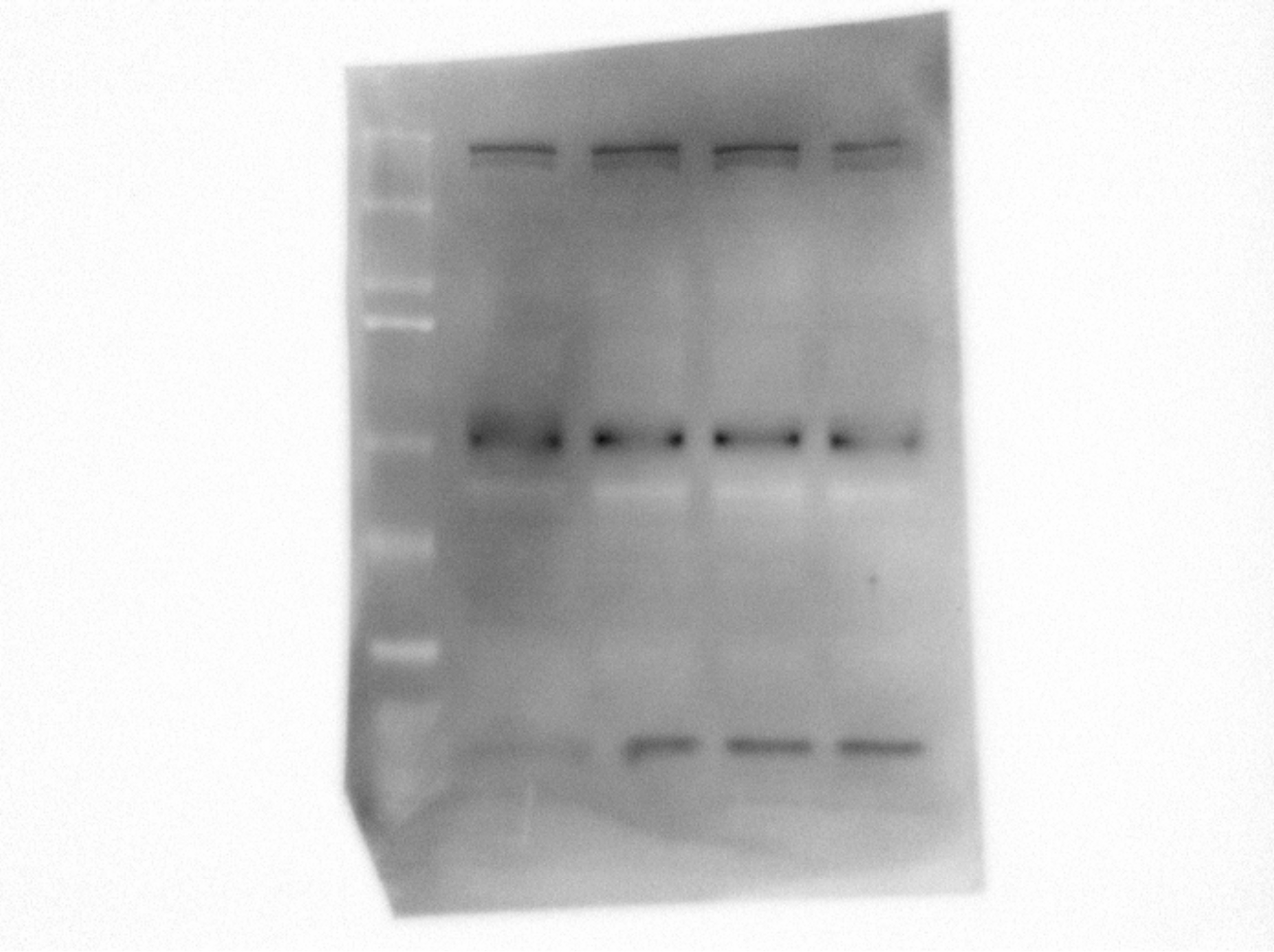

Supplement: Figure 3—source data 2. [file elife-103118-fig3-data2.zip › Figure 3-source data 2/Figure 3D Source Data 2 - A.tif]

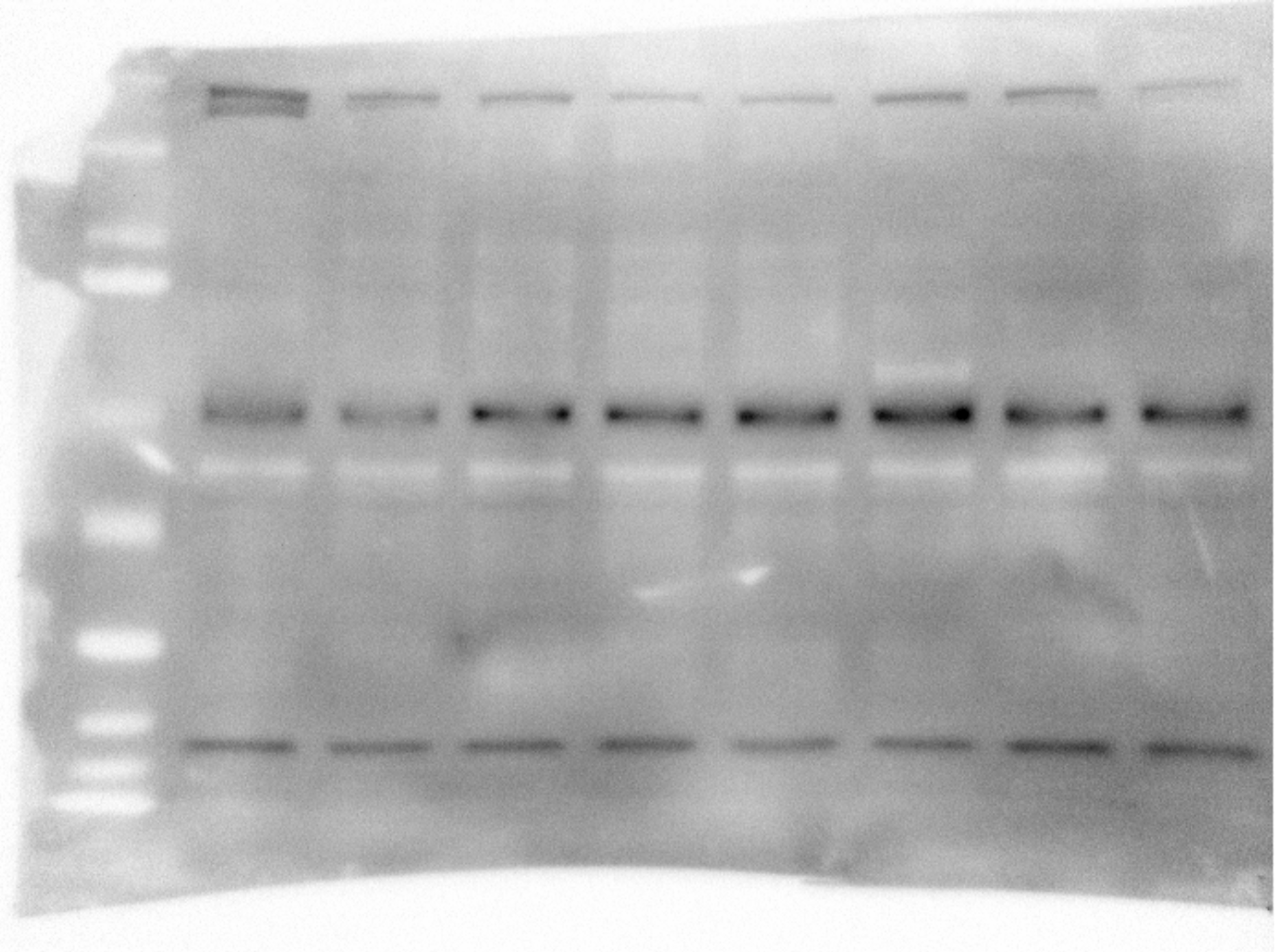

Supplement: Figure 3—source data 2. [file elife-103118-fig3-data2.zip › Figure 3-source data 2/Figure 3D Source Data 2 - BC.tif]
