# Supplementary material for: Age-dependent H3K9 trimethylation by dSetdb1 impairs mitochondrial UPR leading to degeneration of olfactory neurons and loss of olfactory function in Drosophila
Source: eLife. 2026 Mar 26;15:e103118. doi: 10.7554/eLife.103118 (PMC13155754; doi:10.7554/eLife.103118)
Supplement: MDAR checklist [file elife-103118-mdarchecklist1.docx]

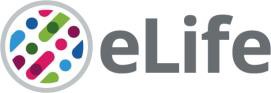

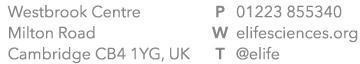


Materials Design Analysis Reporting (MDAR) Checklist for Authors

The [MDAR framework](https://osf.io/xfpn4/) establishes a minimum set of requirements in transparent reporting mainly applicable to studies in the life sciences.

*eLife* asks authors to provide detailed information within their article to facilitate the interpretation and replication of their work. Authors can also upload supporting materials to comply with relevant reporting guidelines for health-related research (see [EQUATOR Network](http://www.equator-network.org/)), life science research (see the [BioSharing Information Resource](http://biosharing.org/)), or animal research (see the ARRIVE Guidelines and the [STRANGE Framework;](https://doi.org/10.1038/d41586-020-01751-5) for details, see *eLife*’s [Journal Policies](https://reviewer.elifesciences.org/author-guide/journal-policies)). Where applicable, authors should refer to any relevant reporting standards materials in this form.

For all that apply, please note where in the article the information is provided. Please note that we also collect information about data availability and ethics in the submission form.

Materials:

| Newly created materials | Indicate where provided: section/figure legend | N/A |
| --- | --- | --- |
| The manuscript includes a dedicated "materials availability statement" providing transparent disclosure about availability of newly created materials including details on how materials can be accessed and describing any restrictions on access. | Methods section, under the subheading "Hsp60::dsRed and Hsc70-5::dsRed Reporter Generation".  This section describes the generation of the new transgenic reporter fly lines. All newly created materials, including the Hsp60::dsRed and  Hsc70-5::dsRed transgenic flies, are available from the corresponding author upon reasonable request. |  |
|  | | |
| Antibodies | Indicate where provided: section/figure legend | N/A |
| For commercial reagents, provide supplier name, catalogue number and [RRID,](https://scicrunch.org/resources) if available. | Methods section, under subheadings "Protein Quantification and western blotting" and "Dissection of adult Drosophila brains and confocal microscopy".  The following commercial antibodies were used:  For Western Blotting (Figure 2A):  Primary Antibody: Rabbit anti-H3K9me3 (Abcam, cat# ab8898), 1:2000 dilution.  Primary Antibody (Loading Control): Mouse anti-Tubulin (Thermofisher, cat# MA1-744), 1:1000 dilution.  Secondary Antibody: HRP-conjugated anti-rabbit IgG (Thermofisher), 1:1000 dilution.  For Immunofluorescence (Figure 4A):  Primary Antibody: Rabbit anti-H3K9me3 (Abcam, cat# ab8898), 1:500 dilution.  Primary Antibody: anti-GFP (Invitrogen), 1:1000 dilution.  Secondary Antibody: Donkey anti-Rabbit 555 (Thermofisher), 1:1000 dilution. |  |

| DNA and RNA sequences | Indicate where provided: section/figure legend | N/A |
| --- | --- | --- |
| Short novel DNA or RNA including primers, probes: Sequences should be included or deposited in a public repository. |  | N/A |
|  | | |
| Cell materials | Indicate where provided: section/figure legend | N/A |
| Cell lines: Provide species information, strain. Provide accession number in repository OR supplier name, catalog number, clone number, OR RRID. |  | N/A |
| Primary cultures: Provide species, strain, sex of origin, genetic modification status. |  | N/A |
|  | | |
| Experimental animals | Indicate where provided: section/figure legend | N/A |
| Laboratory animals or Model organisms: Provide species, strain, sex, age, genetic modification status. Provide accession number in repository OR supplier name, catalog number, clone number, OR RRID. | Methods section, under subheading "Drosophila Strains and Culture".  Species: Drosophila melanogaster.  Sex: Only female flies were used for all experiments.  Age: Flies were aged for experiments and analyzed at multiple time points, typically 0, 15, 30, and 45 days post-eclosion (dpe).  Housing and Husbandry: All stocks were maintained on a standard Drosophila medium at 25°C under a 12:12 hour light:dark cycle.  Genetic Lines and Supplier: All strains were obtained from the Bloomington Drosophila Stock Center (BDSC), Indiana University. The specific lines used, with their catalog numbers (BL#), are detailed in this section and include:  Controls: Wild-type Canton S (BL#64349), RNAi Control (TRIP, BL#35787).  Reporters: UAS-Mito-GFP (BL#8443), UAS-MitoTimer (BL#57323).  Gal4 Drivers: Elav-Gal4 (BL#485), Actin-Gal4 (BL#9431), GH146-Gal4 (BL#30026).  RNAi Lines: crc (BL#25985), dve (BL#26225), ubl (BL#65893), dSetdb1 (TRiP.HMC03152, BL#31352), dSetdb1 (TRiP.HMS04400, BL#58298), Utx (BL#34076), Kdm2 (BL#33699).  Loss-of-function Allele: dSetdb1 lof (egg²³⁵, BL#30566).  Other lines: GH146,GFP (BL#36500). |  |
| Animal observed in or captured from the field: Provide species, sex, and age where possible. |  | N/A |
|  | | |
| Plants and microbes | Indicate where provided: section/figure legend | N/A |
| Plants: provide species and strain, ecotype and cultivar where relevant, unique accession number if available, and source (including location for collected wild specimens). |  | N/A |
| Microbes: provide species and strain, unique accession number if available, and source. |  | N/A |

| Human research participants | Indicate where provided: section/figure legend) or state if these demographics were not collected | N/A |
| --- | --- | --- |
| If collected and within the bounds of privacy constraints report on age, sex, gender and ethnicity for all study participants. |  | N/A |

Design:

| Study protocol | Indicate where provided: section/figure legend | N/A |
| --- | --- | --- |
| If the study protocol has been pre-registered, provide DOI. For clinical trials, provide the trial registration number OR cite DOI. |  | N/A |
|  | | |
| Laboratory protocol | Indicate where provided: section/figure legend | N/A |
| Provide DOI OR other citation details if detailed step-by-step protocols are available. |  | N/A |
|  | | |
| Experimental study design (statistics details) * | | |
| For in vivo studies: State whether and how the following have been done | Indicate where provided: section/figure legend. If it could have been done, but was not, write “not done” | N/A |
| Sample size determination | The manuscript does not describe a pre-experimental power analysis for sample size determination. Sample sizes (n), representing the number of individual animals, cells, or independent experiments, were chosen based on standard practices in the field and are reported for each experiment in the corresponding figure legends and the Methods section "Statistical analysis". |  |
| Randomisation | For each experiment, animals were selected at random from a larger, age-matched, and genetically homogeneous population. No formal randomization protocol was used to allocate animals to specific experimental groups. |  |
| Blinding | All quantifications were performed blindly - see the Methods section  entitled “Statistical analysis”. |  |
| Inclusion/exclusion criteria | Not done |  |
|  | | |
| Sample definition and in-laboratory replication | Indicate where provided: section/figure legend | N/A |
| State number of times the experiment was replicated in the laboratory. | The number of replicates for each experiment is stated in the respective figure legend. Key experiments, such as Western blots (Fig. 2A), were performed in at least 3 independent biological replicates. For imaging experiments, the sample size 'n' typically represents 6-11 individual flies per condition. For behavioral assays, such as the olfactory T-maze (Fig. 3B, Supp. Fig. 5), 'n' represents 3 independent populations of flies tested. |  |

| Define whether data describe technical or biological replicates. | All data describe biological replicates. As stated in the Methods section "Statistical analysis", the sample size 'n' refers to biological replicates. The definition of a biological replicate varies by experiment type: for imaging and immunofluorescence, 'n' represents individual animals (flies) or cells; for Western blots, 'n' represents independent experimental repeats using distinct biological samples; and for behavioral experiments like the olfactory T-maze assay, 'n' represents a distinct population of animals (e.g., a group of 10-15 flies) treated as a single replicate. |  |
| --- | --- | --- |
|  | | |
| Ethics | Indicate where provided: section/submission form | N/A |
| Studies involving human participants: State details of authority granting ethics approval (IRB or equivalent committee(s), provide reference number for approval. |  | N/A |
| Studies involving experimental animals: State details of authority granting ethics approval (IRB or equivalent committee(s), provide reference number for approval. | This study was conducted using the invertebrate model organism Drosophila melanogaster. Research involving invertebrates does not require formal ethics approval by an Institutional Animal Care and Use Committee (IACUC) or an equivalent ethics board. All experiments were performed in accordance with standard laboratory practices and guidelines for the care and handling of Drosophila. |  |
| Studies involving specimen and field samples: State if relevant permits obtained, provide details of authority approving study; if none were required, explain why. |  | N/A |
|  | | |
| Dual Use Research of Concern (DURC) | Indicate where provided: section/submission form | N/A |
| If study is subject to dual use research of concern regulations, state the authority granting approval and reference number for the regulatory approval. |  | N/A |

Analysis:

| Attrition | Indicate where provided: section/figure legend | N/A |
| --- | --- | --- |
| Describe whether exclusion criteria were pre-established. Report if sample or data points were omitted from analysis. If yes, report if this was due to attrition or intentional exclusion and provide justification. | No pre-established exclusion criteria were described in the manuscript. All data generated and analyzed during this study are included in the figures and their corresponding source data files. No data points were omitted from the analysis. |  |
|  | | |
| Statistics | Indicate where provided: section/figure legend | N/A |
| Describe statistical tests used and justify choice of tests. | All statistical analyses are described in the Methods section under the subheading "Statistical analysis". Analyses were performed using GraphPad Prism 9.0. Data are presented as mean ± SEM. The choice of test was based on the experimental design. For comparisons between two groups, Student's t-test was used. For comparisons between multiple groups, one-way or two-way ANOVA was used, followed by either Bonferroni's, Dunnett's, or Tukey's multiple comparisons post-hoc tests. Survival curves were analyzed using the Log-rank (Mantel-Cox) test. The specific test used for each experiment is detailed in the corresponding figure legend. A p-value < 0.05 was considered statistically significant. |  |
|  | | |
| Data availability | Indicate where provided: section/submission form | N/A |
| For newly created and reused datasets, the manuscript includes a data availability statement that provides details for access (or notes restrictions on access). | All newly generated data associated with this study are presented in the main figures, supplementary figures, and the accompanying raw data Excel file provided as Supplementary Material. Reused data from the single-cell RNA-seq analysis is publicly available and cited in the Methods section under "Single Cell RNA-seq Data analysis". |  |
| When newly created datasets are publicly available, provide accession number in repository OR DOI and licensing details where available. |  | N/A |
| If reused data is publicly available provide accession number in repository OR DOI, OR URL, OR citation. | The reused single-cell RNA-seq dataset is publicly available from the Gene Expression Omnibus (GEO) under accession code GSE107451. This is also accessible via the SCope database (http://scope.aertslab.org). |  |
|  | | |
| Code availability | Indicate where provided: section/figure legend | N/A |
| For any computer code/software/mathematical algorithms essential for replicating the main findings of the study, whether newly generated or re-used, the manuscript includes a data availability statement that provides details for access or notes restrictions. | Methods section, under subheadings "Image quantification", "Statistical analysis", and "Single Cell RNA-seq Data analysis". The following software and packages were used for data analysis and are described in the manuscript:  Imaris Software (Bitplane/Oxford Instruments) for 3D image reconstruction and quantification.  GraphPad Prism 9.0 for statistical analysis and graph generation.  SCope (http://scope.aertslab.org) and the “ScopeLoomR” package in R for single-cell RNA-seq data analysis. |  |
| Where newly generated code is publicly available, provide accession number in repository, OR DOI OR URL and licensing details where available. State any restrictions on code availability or accessibility. |  | N/A |

| If reused code is publicly available provide accession number in repository OR DOI OR URL, OR citation. | The reused single-cell RNA-seq dataset was analyzed using the publicly available web tool SCope (http://scope.aertslab.org) and the "ScopeLoomR" package, which is available through standard R programming language repositories. | N/A |
| --- | --- | --- |

Reporting:

The MDAR framework recommends adoption of discipline-specific guidelines, established and endorsed through community initiatives.

| Adherence to community standards | Indicate where provided: section/figure legend | N/A |
| --- | --- | --- |
| State if relevant guidelines (e.g., ICMJE, MIBBI, ARRIVE, STRANGE) have been followed, and whether a checklist (e.g., CONSORT, PRISMA, ARRIVE) is provided with the manuscript. | This study involves in vivo experiments using the model organism Drosophila melanogaster. The reporting of the study design, experimental procedures, and data analysis adheres to the principles of transparency and rigor promoted by community standards, including the relevant recommendations within the ARRIVE (Animal Research: Reporting of In Vivo Experiments) guidelines.  Details regarding animal strains, husbandry, experimental design, and statistical methods are provided throughout the Methods section. This MDAR checklist is being completed to formally document adherence to these community standards as required by the journal. | N/A |

- We provide the following guidance regarding transparent reporting and statistics; we also refer authors to [Ten common statistical mistakes to watch out for when writing or reviewing a manuscript.](https://doi.org/10.7554/eLife.48175)

# Sample-size estimation

- - You should state whether an appropriate sample size was computed when the study was being designed
  - You should state the statistical method of sample size computation and any required assumptions
  - If no explicit power analysis was used, you should describe how you decided what sample (replicate) size (number) to use

# Replicates

- - You should report how often each experiment was performed
  - You should include a definition of biological versus technical replication
  - The data obtained should be provided and sufficient information should be provided to indicate the number of independent biological and/or technical replicates
  - If you encountered any outliers, you should describe how these were handled
  - Criteria for exclusion/inclusion of data should be clearly stated
  - High-throughput sequence data should be uploaded before submission, with a private link for reviewers provided (these are available from both GEO and ArrayExpress)

# Statistical reporting

- - Statistical analysis methods should be described and justified
  - Raw data should be presented in figures whenever informative to do so (typically when N per group is less than 10)
  - For each experiment, you should identify the statistical tests used, exact values of N, definitions of center, methods of multiple test correction, and dispersion and precision measures (e.g., mean, median, SD, SEM, confidence intervals; and, for the major substantive results, a measure of effect size (e.g., Pearson's r, Cohen's d)
  - Report exact p-values wherever possible alongside the summary statistics and 95% confidence intervals. These should be reported for all key questions and not only when the p-value is less than 0.05.

# Group allocation

- - Indicate how samples were allocated into experimental groups (in the case of clinical studies,

please specify allocation to treatment method); if randomization was used, please also state if restricted randomization was applied

- - Indicate if masking was used during group allocation, data collection and/or data analysis
